# Respective Contributions of Instrumented 3D Gait Analysis Data and Tibial Motor Nerve Block on Presurgical Spastic Equinus Foot Assessment: A Retrospective Study of 40 Adults

**DOI:** 10.3389/fneur.2022.862644

**Published:** 2022-05-27

**Authors:** Camille Cormier, Clément Sourisseau, Emmeline Montane, Marino Scandella, Evelyne Castel-Lacanal, Xavier De Boissezon, Philippe Marque, David Gasq

**Affiliations:** ^1^Department of Physiological Explorations, University Hospital of Toulouse, Toulouse, France; ^2^ToNIC, Toulouse NeuroImaging Centre, Inserm, University of Toulouse 3, Toulouse, France; ^3^Department of Physical and Rehabilitation Medicine, University Hospital of Toulouse, Toulouse, France; ^4^Gait Analysis Laboratory, Department of Pediatric Surgery, University Hospital of Toulouse, Toulouse, France

**Keywords:** brain injuries, spinal cord injury (SCI), foot deformities, nerve block, gait analysis, electromyography, muscle spasticity

## Abstract

Spastic equinus foot is a common deformity in neurologic patients who compromise walking ability. It is related to the imbalance between weak dorsiflexion and overactive plantar flexor muscles. To achieve the best functional results after surgical management, the challenge is to identify the relevant components involved in the deformity using several methods, namely, examination in the supine position, motor nerve blocks allowing transient anesthesia of suspected overactive muscles, and kinematic and electromyographic data collected during an instrumented 3D gait analysis. The procedure is not standardized; its use varies from one team to another. Access to gait analysis laboratories is limited, and some teams do not perform motor nerve blocks. When both examinations are available, instrumental data from the instrumented 3D gait analysis can be used to specify muscle targets for motor blocks, but data collected from both examinations are sometimes considered redundant.

This retrospective cohort analysis compared examination in the supine position, temporary motor nerve blocks, and instrumented 3D gait analysis data in 40 adults after brain or spinal cord injuries. Clinical data collected before motor nerve block was not associated with instrumental data to assess calf muscle's overactivity and tibialis anterior function. Improvement of ankle dorsiflexion in the swing phase after tibial motor nerve block was associated with soleus spastic co-contraction during this phase corroborating its involvement in ankle dorsiflexion defects. This study showed the relevance of tibial motor nerve block to remove spastic calf dystonia and facilitate the assessment of calf contracture. It also underlined the need for complementary and specific analyses of the tibialis anterior abnormal activation pattern after motor nerve block to confirm or deny their pathological nature.

## Introduction

Spastic equinus foot (SEF) is a common neuro-orthopedic disorder that compromises walking ability. It results in an imbalance between contracture or spastic overactivity of ankle plantar flexor muscles (triceps surae [TS], flexor digitorum and hallucis longus, and tibialis posterior and peroneus longus) and dorsiflexor muscle paresis (mainly tibialis anterior [TA], secondarily extensor digitorum, and hallucis longus) (1, 2). Paresis of the dorsiflexor muscles is manifested by an activation defect and/or weakness (3). Spastic overactivity can manifest in different ways: spasticity corresponding to an increase in velocity-dependent stretch reflexes, which is clinically manifested by an excessive response to muscle stretching; spastic co-contraction, which is described as inappropriate and stretch-sensitive activation of antagonist muscles during voluntary movement; and spastic dystonia, corresponding to tonic permanent muscle contraction (4). Lack of movement contributes to an increase in connective tissue, leading to an increase in muscle viscosity and contracture corresponding to spastic myopathy, preferentially affecting overactive muscles that are shortened due to the force imbalance in the agonist/antagonist couple (3).

By improving ankle dorsiflexion (ADF) during swing and stance phases, neuro-orthopedic surgery of SEF improves walking speed and almost all spatiotemporal parameters and reduces the need for orthoses (5–7). Many techniques are possible, but at present, it is difficult to choose as the assessment tools used in the literature are heterogeneous (8), and above all, because there are several suitable techniques depending on the pathophysiology of the deformity (7, 9). Neurotomy is preferable in patients with isolated spastic overactivity of the calf, whereas musculotendinous lengthening is preferable in the case of calf contracture (9). In the presence of ankle dorsiflexor paresis, it may be possible to improve ADF during swing and/or to correct excessive varus by tendon transfer of an active or even an overactive muscle (10), either through active contraction of the transferred muscle or through a passive effect linked to tendon-muscle complex tensioning. Therefore, the challenge of surgical management of SEF is the quality assessment that enables the identification of the different components involved in the foot deformity in order to achieve the best functional results (8, 9).

SEF can be assessed by different methods. Clinical assessment, at least assisted by videos for gait analysis, is the first essential but often insufficient step. Quantified gait analysis (3D-IGA), which is the gold standard for gait disorder assessment, comprises a synchronized collection of kinematic, kinetic, video, and dynamic electromyographic (EMG) data in a standardized setting, but this is not readily available to all physicians. Its impact on therapeutic decision-making leads to changes in the surgical procedures performed in 52–89% of cases, providing greater confidence in surgery (10). Temporary motor nerve block (MNB) that consists of reducing the contraction of a muscle by injecting an anesthetic around a nerve (e.g., popliteal tibial nerve) or the muscle motor nerve branches results in decreased contraction of the targeted muscles and is effective in replicating post-neurotomy results (11).

The challenge to achieve the best functional therapeutic results in the SEF management is to identify the respective implications of dorsiflexor paresis and plantar flexor overactivity and contracture in the deformation (9). The assessment is not standardized (12) and may be based on clinical data, sometimes supplemented by transient chemical denervation, or on instrumental data collected during a 3D-IGA. The main objective was to quantify the strength of the association between 1) the data issue from 3D-IGA and the data collected before and after MNB during 2) supine analytical examination, and 3) visual gait pattern analysis. The secondary objective was to determine the clinical and instrumental factors predictive of swing ADF improvement.

## Method

This monocentric retrospective observational cohort study was conducted from April 2016 to March 2020 in a neurorehabilitation center (Toulouse University Hospital, France). The study focused on patients with SEF due to brain or spinal cord injury, assessed by both 3D-IGA and tibial MNB.

Patients received information about anonymized data collection, and the study was registered (registration number: RnIPH 2020-59) and covered by the MR-004 (CNIL number: 2206723 v 0) according to French ethics and regulatory law (Public Health Code).

### Participants

The inclusion criteria were as follows: 1) central neurological pathology responsible for SEF; 2) gait assessment by 3D-IGA carried out before the MNB; 3) temporary calf MNB targeting truncal tibial nerve block or gastrocnemius motor branches and/or soleus superior motor branch and/or tibialis posterior motor branch and/or selective intramuscular anesthetic blocks; and 4) at least 18 years of age at the time of consent.

The exclusion criteria applied to patients who i) refused to allow their data to be used for research purposes; ii) had benefited from MNB other than that indicated above; and iii) had undergone calf surgery between 3D-IGA and MNB.

### Kinematic and Electromyographic Data

The 3D-IGA was performed in the University Hospital Gait Laboratory. Kinematic data were collected using an optoelectronic system of eight high-resolution infrared cameras (Vicon, Oxford Metrics, UK) with passive markers positioned according to the modified Hayes model (13). Kinematic data were averaged for each patient from data from 11 gait cycles. EMG data were collected with a 16-channel wireless surface electrode dynamic electromyography system (Wave Plus Wireless sEMG system, Cometa, Italy) without any normalization procedure. No preprocessing was applied to the EMG data. All these data were expressed from 0 to 100% over the gait cycle.

### Tibial Nerve Block

The MNB muscle targets were chosen considering both the instrumental data and the clinical and anamnestic data collected just before the 3D-IGA. MNB was administered at a distance from the 3D-IGA, during the second day of hospitalization allowing the interpretation of the data collected during the 3D-IGA. In accordance with current recommendations (14), selective blocks of tibial motor nerve branches to the gastrocnemii and/or soleus and/or tibialis posterior were administered under electrostimulation combined with ultrasound guidance. Intramuscular blocks and nonselective tibial nerve blocks were administered in the popliteal fossa under exclusive ultrasound guidance. The use of a long half-acting anesthetic (ropivacaine) prolonged the effect for a more reliable subjective self-assessment. We documented the following potential adverse effects of MNB: persistent pain, persistent sensory or motor disorder, and hematoma.

### Video Camera Setting

The spontaneous speed gait was filmed before and after the MNB in a dedicated room equipped with two high-definition and high-frequency (120 fps) fixed cameras: one in the front/back for frontal plane assessment and one in the profile for sagittal plane assessment. The pre-post videos were analyzed by the Kinovea^®^ software, allowing simultaneous and synchronized viewing and slow motion. Qualitative judgment was made by three independent evaluators.

### Clinical and Instrumental Data

The descriptive data collected were age (years), gender (male/female), pathology, the period between 3D-IGA and MNB (months), modified Functional Ambulation Classification scale score (15), and gait speed (m.s^−1^).

The same investigator (referring PRM physician) performed the pre- and post-MNB clinical evaluations under identical conditions. The results from the analytical clinical examination were as follows:

- Passive ankle range of motion (in degrees) was measured in supine position with both knees flexed and extended;
- Spasticity was measured with the Tardieu scale (muscle reaction from 0 to 4) (4, 9) according to the SOFMER and SFAR recommendations (14) for the following muscles: the TS, both knee-flexed (mainly soleus) and knee-extended (gastrocnemii and soleus), tibialis posterior, and peroneus longus muscles;
- Motor strength, measured with the Held scale (16) for the TA, tibialis posterior, and peroneus longus muscles (preferable to the MRC scale as it does not consider the range of motion, which can reflect the presence of spastic co-contractions) or the Medical Research Council (MRC) scale for triceps surae (3/5 corresponding to the possibility of standing on the tip of the toes in unipedal support);
- Motor TA selectivity was measured using the Boyd scale (17);
- The occurrence of distal sensory and/or vasomotor disorders after the MNB.

**Figure 1:**
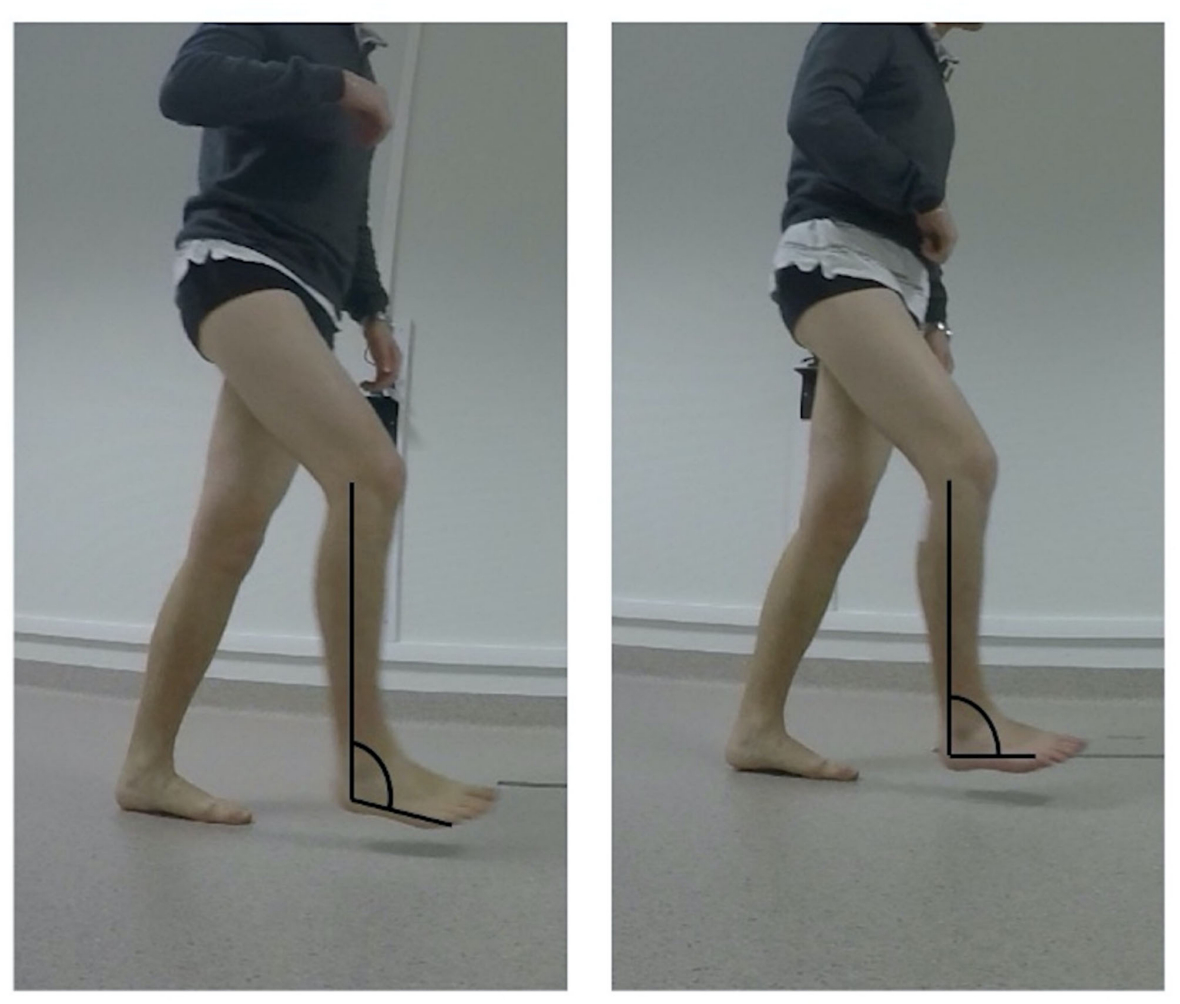
Illustration of improved ankle dorsiflexion confirmed by pre- (on the left) and post- (on the right) motor nerve block comparison in the sagittal plane video.

The characteristics of the gait pattern were assessed before and after the MNB by qualitative video analysis as described in the Edinburgh visual gait score (18), including the collection of the following criteria: maximal ADF limitation during the stance (<5°) and the swing phase (<0°) and hind footvarus during stance. The following criteria were blindly assessed by three operators from videos recorded in the sagittal plane (Figure 1): Improvement in active ADF after MNB was defined as either an improvement in the maximum active ADF during swing or an improvement in the sagittal position of the foot at the initial contact; visible protrusion of the TA tendon during swing was reported.

The data collected during the patient interview were as follows:

- Patient's complaints about walking such as foot catching, lateral ankle twist, or toe claw before QGA;
- Subjective improvement in the gait comfort reported by the patient after MNB was evaluated with a binary response question.

**Figure 2:**
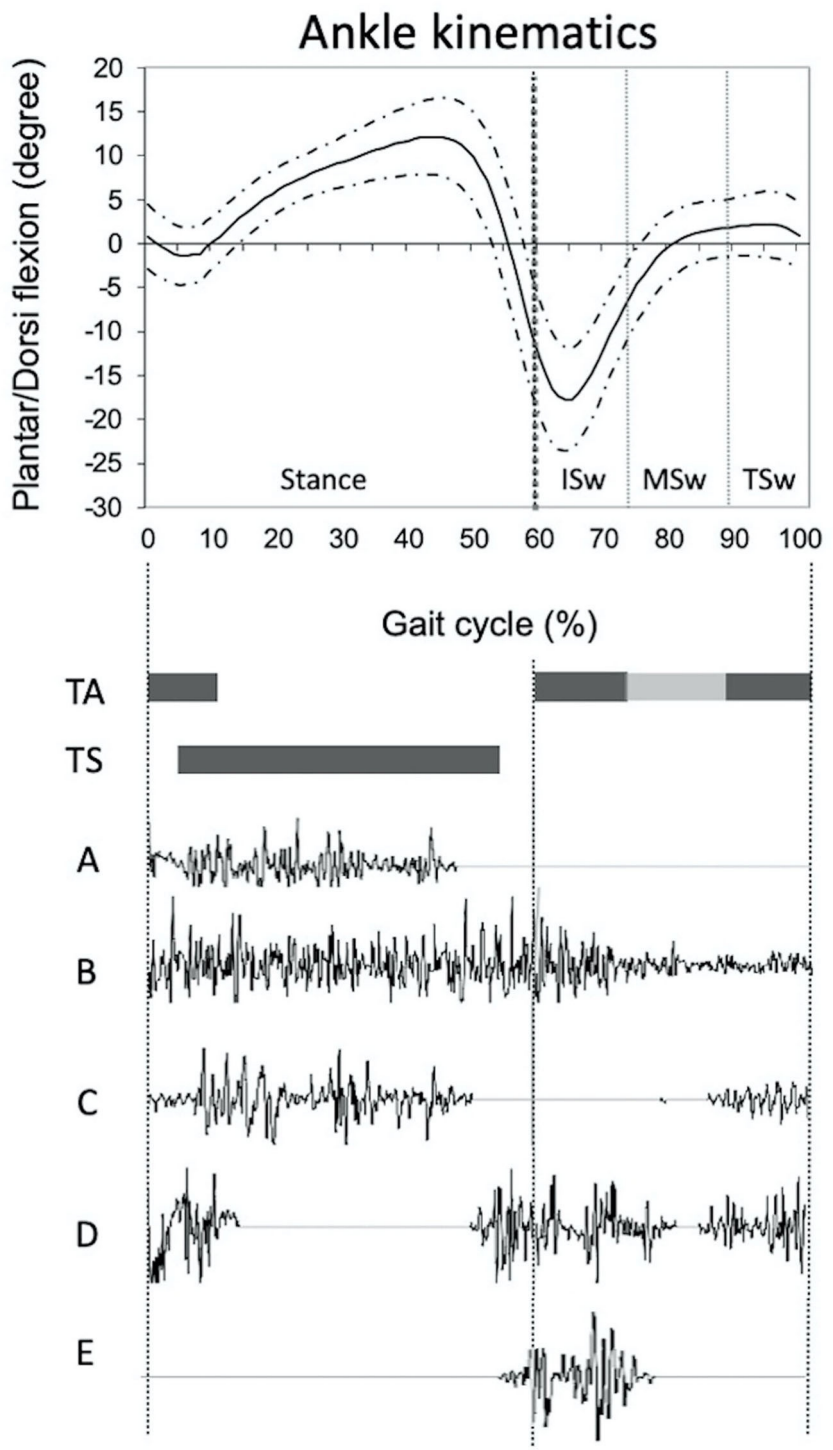
Illustration of ankle physiological kinematic curve (mean value, continued curve ± one standard deviation, dotted curve) and physiological timing of activation (black cases) of tibialis anterior and triceps surae muscles. Description of the different EMG patterns of triceps surae **(A–C)** and tibialis anterior **(D,E)**: **(A)** Physiological, no activation in swing phase; **(B)** Overactivity during initial swing phase; **(C)** Overactivity during terminal swing phase; **(D)** Physiological activation with activation in the swing phase, before the heel strike and prolonged at the beginning of the stance phase; **(E)** Abnormal pattern characterized by a loss of second activation. ISw, initial swing; MSw, mid swing; TA, tibialis anterior; TS, triceps surae; TSw, terminal swing.

Instrumental data extracted from 3D-IGA were peak ADF during stance and swing phases, and EMG data from the medial gastrocnemius, soleus, peroneus longus, and TA. The swing phase was divided into three periods as follows: “early-mid swing” from toe-off to the time when the swinging foot was opposite the stance foot, “mid-swing” from the end of the previous phase to when the tibia of the swinging limb was vertical, and “terminal swing” from the end of mid-swing to foot strike (2). The EMG patterns of calf and peroneus longus muscles were described in three categories: physiological activation, overactivity during early and/or mid-swing (with or without terminal swing overactivity), and isolated overactivity during terminal swing (Figure 2). EMG patterns of TA were described as “physiological,” “abnormal first activation” (i.e., loss or decreased the first activation in early swing), and “isolated abnormal second activation” (i.e., loss or decreased second activation in terminal swing and initial contact). Three operators performed a blind qualitative analysis of EMG data from 5 to 6 gait cycles to minimize bias in characterizing EMG patterns.

#### Efficiency of Motor Nerve Blocks

**Figure 3:**
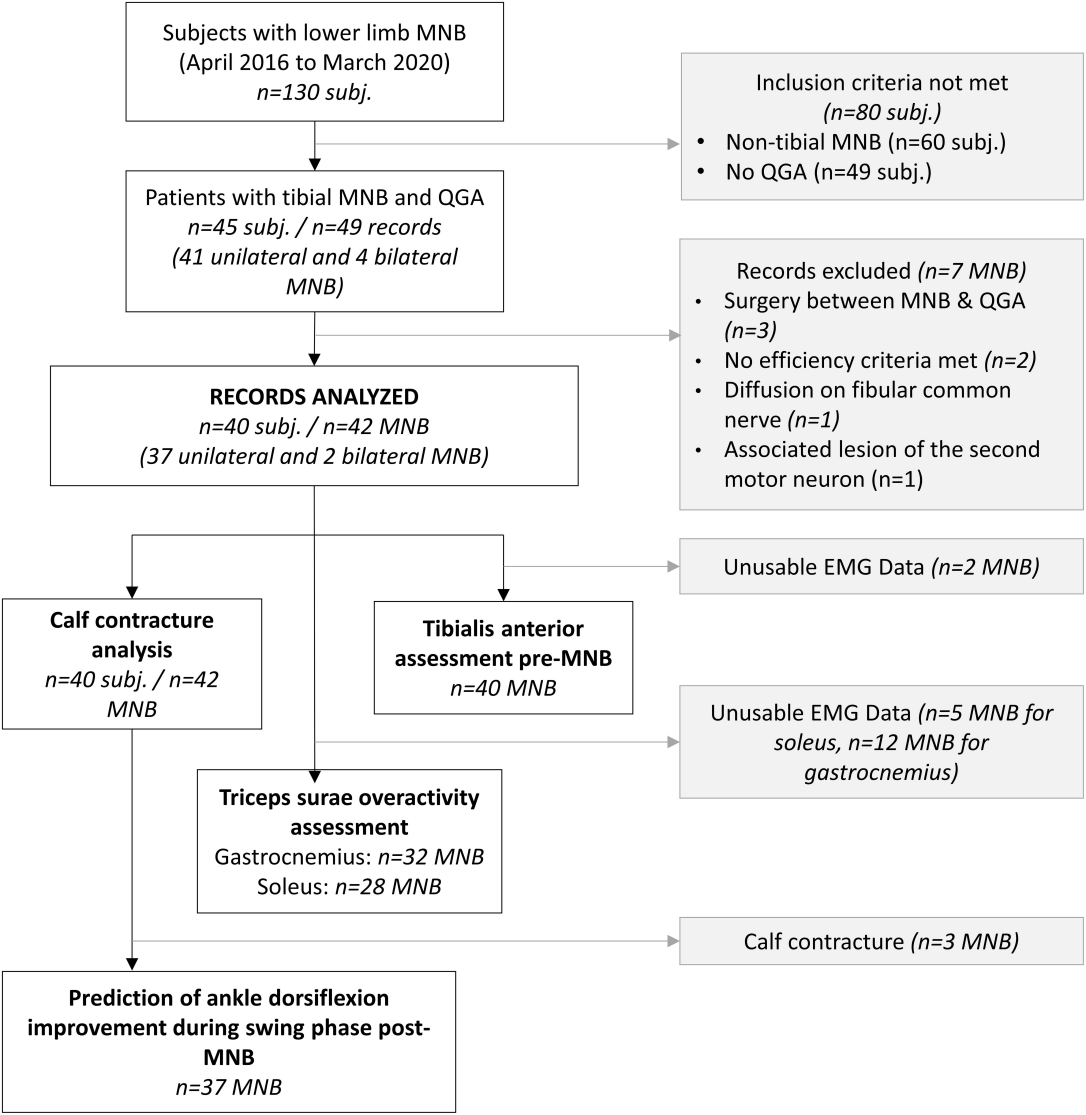
Flowchart (3D-I3D-IGA, instrumented 3D gait analysis; MNB, motor nerve block; subj, subject).

The following criteria were applied to each targeted muscle: a reduction of at least 1 point in the MRC or Held score and/or a reduction of at least 1 point in the Tardieu subscale. The absence of at least 1 of these efficiency criteria led us to consider MNB ineffective, and it was removed from the analyses (see flowchart in Figure 3).

### Statistical Analyses

Statistical analyses were performed using the R software (version 4.1.0, R Foundation, Vienna, Austria) and the R Studio interface (version 1.4.1717).

Demographic data were described in terms of number and percentage for categorical variables, while continuous data were described in terms of mean, standard deviation, minimum, and maximum values.

We examined the relationship between pre- and post-MNB clinical data and instrumental data with odds ratio and the estimated 95% confidence interval (OR [95% CI]), interpreting these as effect size indicators and deeming them of interest and relevance when > 4 (19). Although the amplitude of the sample size has little impact on the effect size, the confidence interval is reduced with an increase in the statistical power of the study (20). Consequently, in a pilot study with low statistical power, it is possible to discuss the amplitude of size effect even if the confidence interval includes the zero value. We examined the positive and negative predictive values (PPV and NPV), particularly when it was not possible to calculate the OR (a headcount of 0).

We studied associations between:

- The limitation of maximal ADF was assessed in the supine position (<0°) both with knee-flexed (KF) and knee-extended (KE), and it was also assessed during the stance phase with 3D-IGA (<0°) and with EVGS (<5° of ADF).
- Pathological EMG-activation of gastrocnemii and soleus during the swing phase (i.e., spastic co-contraction or spastic dystonia) and spasticity (defined by a Tardieu score of at least 2 pre-MNB).
- Physiological EMG-activation of TA and both good strength (defined by a Held score ≥ 4) and good selectivity of recruitment (Boyd score = 4) post-MNB.
- Improvement of ADF during the swing phase post-MNB (exclusion of cases with an ADF <0° with KF post-MNB) on one hand, and TA strength (Held) and/or selectivity (Boyd), dynamic EMG patterns of medial gastrocnemius and soleus on the other hand.

## Results

### Participants

A total of 126 lower limb MNBs were administered and finally 42 MNBs administered to 40 subjects were included in the analysis (refer to the flowchart in Figure 3 for details). All patients had previously received botulinum toxin injections in the calf muscles, but both 3D-IGA and MNB occurred outside the efficacy period, i.e., when the patient no longer feels the effects of the botulinum toxin on the functional objectives and at least 4 months after the last injection. The mean time from 3D-IGA to MNB was 7.12 [4.56–9.68] months. Table 1 presents the demographic data of the population. The instrumental and clinical data collected for each record are presented in the supplemental materials (Supplementary Table S1).

**Table 1:** Detailed population characteristics (*n* = 41).

Population. *n (%)*	40 (100)
Ischemic stroke	12 (30)
Hemorrhagic stroke	10 (25)
Traumatic brain injury	4 (10)
Cerebral palsy	7 (17)
Spinal cord injury	3 (8)
Brain tumor	2 (5)
Hereditary spastic paraparesis	2 (5)
Age. years. *Mean [95% CI]; min to max values*	38 (21–31); 18 to 74
Self-selected walking speed. m.s^−1^. *Mean [95% CI]; min to max values*	0.69 [0,59–0,77]; 0.12 to 1.29
Modified FAC (0–3/4/5/6/7/8). n	0 / 3 / 2 / 22 / 6 / 7
Period between 3D-IGA and MNB in months. *Mean [95% CI]; min to max values*	7 (5–9, 32); 0 to 45

*FAC, Functional Ambulation Classification; MNB, motor nerve block; 3D-I3D-IGA, instrumented 3D gait analysis; 95% CI, 95% confidence interval*.

The mean spontaneous gait speed was 0.68 ± 0.30 m.s^−1^. The main disorders were foot catching (*n* = 36/42; 86%) and lateral ankle twist (*n* = 30/42; 71%). Qualitative video assessment using EVGS highlighted the limitation of ADF for 22/42 (52%) both in stance and swing phases and varus during stance for 12/42 (29%) feet.

### MNB Procedure Characteristics

In total, 24 (57%) MNB procedures specifically targeted gastrocnemii, 31 (74%) the soleus, 30 (71%) the tibialis posterior, 6 (15%) the flexor digitorum longus, 5 (12%) the flexor hallucis longus, and 1 (2%) the fibularis longus. A total of 40 (95%) MNB met the criteria for complete analytical effectiveness (disappearance of spasticity and/or full paresis in the targeted muscles). After MNB, the TA Held score increased in 9 (64% of subjects with Held score <4 pre-MNB) cases, and the Boyd score increased in 15 (35% of subjects with Boyd score <4 pre-MNB) cases.

In 23 (55%) cases, MNB caused sensory disorders, indicating anesthesia or diffusion of the anesthetic product in the tibial nerve trunk. In 6 (14%) cases, MNB caused calf pain in the ensuing days, without any other obvious complication.

### Multiple Approaches to Assess Calf Contracture

Supine ADF assessment pre-MNB was ≤ 0 ° for 21 patients with KF and 35 feet with KE and increased post-MNB for 14 (67%) and 22 (61%) patients, respectively (refer to Table 2). No clinical variable (including qualitative video assessment) was associated with equinus during the stance phase on 3D-IGA except on post-MNB equinus KF in supine position [OR = 8.00 (1.19–53.93)] (refer to Table 3).

**Table 2:** Clinical and instrumental assessment of equinus, triceps surae overactivity, and tibialis anterior functionality.

**Assessment modality**	**Pre-MNB**	**Post-MNB**
Maximal ADF to assess equinus (*n =* 42)		
3D-IGA: ADF ≤ 0°during stance phase	15 (37%)	
Video: ADF ≤ 5° during stance phase	22 (51%)	
Supine ADF ≤ 0°KF	20 (50%)	6 (15%)
Supine ADF ≤ 0°KE	35 (87%)	22 (55%)
Soleus overactivity (*n =* 37)		
3D-IGA: early-mid swing overactivity	9 (24%)	
3D-IGA: isolated terminal swing overactivity	9 (24%)	
Spasticity KF (Tardieu scale ≥ 2)	28 (76%)	
Medial Gastrocnemius overactivity (*n =* 27)		
3D-IGA: early-mid swing overactivity	15 (56%)	
3D-IGA: isolated terminal swing overactivity	8 (30%)	
Spasticity KE (Tardieu scale ≥ 2)	16 (59%)	
Tibialis anterior functionality (*n =* 40)		
3D-IGA: abnormal pattern	28 (72%)	
3D-IGA: isolated abnormal terminal swing activation	27 (67%)	
Video: no protrusion of TA tendon visible	3 (7%)	
Held score <4	15 (37%)	11 (27%)
Boyd score <4	30 (75%)	24 (60%)

*ADF, ankle dorsiflexion; EVGS, Edinburg Visual Gait Score; KE, knee extended; KF, knee flexed; MNB, motor nerve block; 3D-I3D-IGA, instrumented 3D gait analysis*.

**Table 3:** Associations between clinical, instrumental, and post-MNB data studied with odds ratios, positive, and negative predictive values.

	**OR [IC95]**	**PPV**	**NPV**
Equinus assessment			
Video / 3D-IGA	4.00 [1.01–15.87]	50%	80%
Supine assessment KF pre-MNB / 3D-IGA	2.24 [0.36–13.78]	19%	90%
Supine assessment KE pre-MNB / 3D-IGA	*NC*	17%	100%
Supine assessment KF post-MNB / 3D-IGA	8.00 [1.19–53.93]	50%	89%
Supine assessment KE post-MNB / 3D-IGA	2.24 [0.36–13.78]	67%	53%
Soleus, spasticity versus overactivity			
Spasticity KF pre-MNB / 3D-IGA (early–mid swing overactivity)	0.44 [0.08–2.36]	21%	62%
Spasticity KF pre-MNB / 3D-IGA (terminal swing overactivity)	1.11 [0.19–6.64]	24%	77%
Medial gastrocnemius, spasticity versus overactivity			
Spasticity KE pre-MNB / 3D-IGA (early–mid swing overactivity)	0.09 [0.01–0.87]	6%	57%
Spasticity KE pre-MNB / 3D-IGA (terminal swing overactivity)	1.67 [0.32–8.74]	31%	79%
Tibialis anterior functionality			
Video (no protrusion of tendon visible) / 3D-IGA (abnormal pattern)	*NC*	100%	32%
Held score <4 post-MNB / 3D-IGA (abnormal pattern)	2.78 [0.51–15.26]	83%	36%
Boyd score <4 post-MNB / 3D-IGA (abnormal pattern)	3.50 [0.85–14.34]	80%	47%
Video / Held score <4 post–MNB	5.40 [0.44–66.29]	67%	73%
Video / Boyd score <4 post–MNB	1.22 [0.11–14.69]	67%	38%
Prediction of post-MNB improvement of ADF during swing phase			
3D-IGA (physiological pattern) / ADF improvement	1.46 [0.34–6.34]	45%	64%
Held score > 4 post–MNB/ ADF improvement	1.97 [0.41–9.51]	46%	70%
Boyd score > 4 post-MNB / ADF improvement	1.03 [0.25–4.30]	42%	59%
Visible protrusion of TA tendon / ADF improvement	*NC*	46%	100%
3D-IGA (soleus early-mid swing overactivity) / ADF improvement	4.72 [0.86–26.04]	63%	74%
3D-IGA (soleus terminal swing overactivity) / ADF improvement	0.46 [0.09–2.25]	25%	58%
Spasticity KF / ADF improvement	1.12 [0.17–7.39]	33%	67%
3D-IGA (medial gastrocnemius early-mid swing overactivity) / ADF improvement	3.50 [0.46–26.61]	60%	70%
3D-IGA (medial gastrocnemius terminal swing overactivity) / ADF improvement	*NC*	0%	50%
Spasticity KE / ADF improvement	0.48 [0.09–2.52]	29%	55%

*ADF, ankle dorsiflexion; EVGS, Edinburg visual gait score; KE, knee extended; KF, knee flexed; MNB, motor nerve block; 3D-I3D-IGA, instrumented 3D gait analysis; NC, non-calculable*.

### Association Between Triceps Surae Spasticity and Overactivity

Dynamic EMG data during the swing phase was usable for 30 (71%) and 37 (88%) MNB for the medial gastrocnemius and the soleus, respectively (refer to Table 2). We found no association between spasticity and dynamic overactivity (i.e., spastic co-contraction) of the soleus (refer to Table 3).

### Tibialis Anterior Function

We examined the association between four modalities of TA assessment of the 40 MNB with interpretable TA EMG data (refer to Table 2). We found no significant association between physiological TA EMG and clinical data, except an excellent PPV (100%) with no visible protrusion of TA tendon and an abnormal EMG pattern of TA, but concerning 3 patients only (refer to Table 3). Furthermore, a strong association was established between loss of terminal swing TA activation and non-taligrade initial contact at foot-strike [OR = 8.33 (1.60–43.29)].

### Post-MNB Improvement of ADF During the Swing Phase

An increase in ADF during the swing phase has been noted in 17 (43%) of the 39 records for which the ADF KF was ≥ 0° after the MNB. It was associated with subjective improvement [OR = 11.25 (1.25–120.63)].

Table 3 presents relationship between EMG, clinical variables, and swing ADF improvement post-MNB. More relevant OR were found with early and/or mid-swing overactivity of the soleus [OR = 4.72 (0.86–26.04)].

## Discussion

This study confronted clinical data before and after MNB with instrumental data in order to better identify their respective contributions in the presurgical assessment of the SEF. The results highlight that the data collected in the supine position cannot be extended to what happens during movement and are in favor of a negative impact of spastic co-contraction of the soleus on active ankle dorsiflexion.

This study also allowed us to summarize the advantages and limitations of the various clinical and instrumental approaches that can be used to assess SEF (Table 4), highlighting their complementarity.

**Table 4:** Respective advantages and limits of methods used to assess spastic equinus foot.

	**Supine examination and video**	**Clinical assessment pre- and post-motor nerve block**	**Instrumented 3D Gait Analysis**
**Calf contracture**			
*Modalities*	Maximal passive ankle dorsiflexion (knee-flexed and extended) and during stance	Post-MNB maximal ankle dorsiflexion in supine position (knee-flexed and extended) and during stance	Maximal ankle dorsiflexion during stance phase
*Advantages*	Distinction between gastrocnemii and soleus involvement	Distinction between gastrocnemii and soleus contracture No risk of underestimation	Good reliability
*Limits*	Poor accuracy Underestimation in case of calf spastic dystonia and potentially in case of spastic myopathy (in the absence of evaluation under load)	Moderate accuracy and potential underestimation in case of spastic myopathy (in the absence of evaluation under load)	Underestimation in case of calf spastic dystonia Difficulty to differentiate soleus from gastrocnemius contracture due to variability in knee position Overestimation due to measurement error with monosegmental models
**Tibialis anterior functionality**			
*Modalities*	Strength (Held scale) and command selectivity (Boyd scale) Ankle dorsiflexion during swing Visible protrusion of tibialis anterior tendon during swing	Post-MNB strength (Held scale) and command selectivity (Boyd scale) Post-MNB ankle dorsiflexion during swing	Dynamic EMG of the tibialis anterior
*Advantages*		No underestimation in case of spastic overactivity	Contributory in case of calf contracture
*Limits*	Not very contributive in case of calf contracture Underestimation in case of calf co-contraction Not valid because of automatic-voluntary dissociation Poor reliability of tibialis anterior tendon protrusion during swing Poor reliability of visual estimation of ankle dorsiflexion during swing	Not very contributive in case of calf contracture Poor accuracy of the visual estimation of active ankle dorsiflexion during swing Not predictive of dynamic activation because of automatic-voluntary dissociation	No assessment of activation intensity if no normalization procedure Missing data due to poor EMG data quality
**Calf muscle overactivity**			
*Modalities*	Spasticity Ankle dorsiflexion during swing	Improvement of ankle dorsiflexion during swing after the block	Dynamic EMG of the triceps surae
*Advantages*		Distinction of the different muscles involved with selective motor nerve blocks (including tibialis posterior, fibularis longus, flexor digitorum longus and flexor hallucis longus)	Contributory in case of calf contracture Distinction between gastrocnemii and soleus
*Limits*	No relation between spasticity and spastic co-contraction Poor accuracy of the visual estimation of ankle dorsiflexion during swing Non-contributory in case of posterior compartment contracture No distinction of the different muscles involved	Poor accuracy of the visual estimation of ankle dorsiflexion during swing Non-contributory in case of calf contracture No distinction of the different muscles involved in truncal block or in case of diffusion during selective blocks	No information about the tibialis posterior, the flexor longus digitorum and flexor hallucis longus unless implanted EMG is used Missing data due to poor EMG data quality
**Complementary information**			
*Advantages*		Evaluation of the reducibility of frontal plane deformities often associated with equinus	Quantification of the other components of the shortening defect and its compensations
*Limits*			Low reliability of associated ankle-foot anomalies in the frontal plane with conventional models

### MNB Is a Safe Procedure

Our results confirmed that MNB is a safe procedure as no permanent pain, sensory disorder, muscle deficit, or hematoma was observed. This was previously described by Filipetti *et al*. who reported only 5 painful procedures, 2 hematomas, and no severe complications after 815 MNB procedures (33).

### Prediction of Calf Muscle Contracture: The Role of Spastic Dystonia

Our results confirm the poor effectiveness of supine examination to assess calf contracture since a limitation of maximal ADF during the stance phase was not associated with the limitation of passive ADF during the supine examination and was not associated with ADF assessed after MNB. It is likely that passive ADF was underestimated by the presence of spastic dystonia, mimicking contracture, or by spastic myopathy (4). The association between the limitation of maximal ADF during the stance phase and the limitation of maximal passive ADF during the supine examination post-MNB was better, especially with KF, which can be explained by the suppression of spastic dystonia (9, 33). However, the positive predictive value was low, possibly because of the ineffectiveness of MNB on spastic myopathy. Finally, this could also be explained by the imprecise measurement of ADF with monosegmental kinematic models in the presence of frontal deformities of the foot, with a potential overestimation of 7° (34). It could also be explained by the variability in kinematic patterns used to compensate for the lack of forward progression, such as knee recurvatum, which involves maximal stretching of the gastrocnemii (2, 35). Consequently, instrumental measurement of ADF during gait is not satisfactory to evaluate calf contracture, which could be interpreted in conjunction with clinical examination and video to clarify mechanisms involved in ankle/foot deformities (35–37).

### Consequences of Spasticity and Spastic co-Contraction of the Triceps Surae on Active ADF

During an early swing, the TA muscle contracts concentrically, allowing the ankle to perform a rapid dorsiflexion movement, bringing the ankle to 0° for foot clearance (2, 37). Overactivity of the calf muscles, especially the triceps surae, during this period corresponds to spastic co-contraction (4) involved in limiting active movement (38) by reversing dorsiflexor torque to plantar-flexor torque (39). This observation, described as a “stretch-sensitive paresis” (4), was noted for 26% of the cases in a hemiplegic population of 18 subjects (39). Our findings of a strong and significant association between spastic co-contraction of the soleus and an increase in ADF post-MNB, and to a lesser extent of the medial gastrocnemius, are consistent with the literature. This is also coherent with the major implication of soleus overactivity in SEF, which came to light from examining the ratio between the maximum amplitude of the H reflex and the maximum amplitude of the M response (40). Selective MNB decreased this ratio in the case of the soleus muscle with no change in the medial gastrocnemius (41). Compared to gastrocnemius branches, selective soleus MNB also demonstrated a more significant improvement in terms of stretch reflex scores, gait parameters, and patient self-reported improvements (42), and sometimes isolated soleus neurotomies were sufficient to induce a persistent decrease in the triceps surae stretch reflex score (43). Conversely, terminal swing TS overactivity was not associated with an improvement in the ADF after MNB. It is possible that this premature activation reflects a compensatory phenomenon allowing the body weight to be absorbed in foot strike (21, 22). No relation was found between an improvement in ADF post-MNB and TS spasticity either, highlighting that spasticity assessment does not predict what happens during movement (4, 38).

### Need for Improvement in the Assessment of the Tibialis Anterior Function

Our results confirmed that the different TA function assessment modalities were not equivalent. The supine evaluation, before or after MNB, which is an assessment of the strength and selectivity of motor control, was not related to the quality of temporal activation during the swing phase, which indicates an automatic-voluntary dissociation. None of these parameters was predictive of improved ADF post-MNB. In addition, the presence of a visible protrusion of TA tendon on the video also appears unreliable, as it was not related to any of the above criteria.

The poor predictive value of TA EMG activation during the swing phase was previously shown by Burridge et al. in a cohort of 112 patients, among whom more than 50% had a limitation in dorsiflexion speed despite TA activation without triceps surae overactivity (23). In our study, this could be explained by either spastic myopathy, overactivity of plantar flexor muscles not evaluated by dynamic EMG (lateral gastrocnemius, tibialis posterior, fibularis longus), or by the lack of activation of accessory levators (extensor digitorum longus and extensor pollicis longus) (24). In any event, this indicates the need for further studies to analyze a possible modification in TA activation pattern before and after MNB or even to compare the envelopes of EMG signals.

Finally, in our population, there was no improvement in ADF for 4 patients during the swing phase, although they all had a physiological EMG TA pattern with a high Held score (4/5) and no calf contracture. Moreover, 3 had a tibialis posterior MNB, excluding the involvement of this muscle in persistent ADF deficit, but all 4 patients had a very slow walking speed <0.5 m.s^−1^ (mean = 0.31 m.s^−1^) compared to the 6 patients whose ADF increased (walking speed > 0.5 m.s^−1^, mean = 0.67 m.s^−1^). The impact of walking speed on gait kinematics and kinetic parameters has already been demonstrated (36), and this major factor should always be included in the interpretation of instrumental data.

### Significance of a Tibialis Anterior Activation Defect During the Terminal Swing and Early Stance

TA normally activates strongly after heel strike, which controls plantar flexion, and then again at toe-off to lift the foot clear of the ground, peaking during early swing (23). After a stroke, the initial contact activation of TA is particularly low compared to healthy subjects (25). However, the significance of the second abnormal activation of the TA muscle remains uncertain. Our results highlighted no pejorative values in this pattern because it was not associated with a limited ADF during the swing phase after MNB.

Conversely, we found a strong association between abnormalities in the position of the foot at initial contact and an activation defect at the end of the swing phase [OR = 8.33 (1.60–43.29)] that could reflect more of a consequence of the equinus gait than a primitive gait abnormality. This hypothesis is supported by some elements in the literature: several articles have reported decreased activation of the TA during early stance in healthy children fitted with an orthosis simulating an equinus (21), in healthy adult subjects simulating toe-walking, and in adult cerebral palsy subjects (22). Therefore, an alteration in this pattern could reflect either a primitive anomaly that explains the ADF defect during this phase, or a compensatory anomaly linked to the absence of the need for eccentric contraction when the initial contact is not taligrade. In the literature, three studies investigated TA activation patterns before and after SEF foot surgery: They showed no significant change in activation patterns, but the analysis was global, as patterns were only characterized as in phase/out of phase (26) or as continuous, phasic premature and/or prolonged, or physiological (27). Keenan et al. showed the appearance of a phasic activation pattern in 6 of the 16 patients who showed continuous activation before surgery (28). The results of our study are insufficient to prove a causal association, and only a complementary analysis with dynamic EMG before and after MNB or equinus foot surgery, to specifically identify the persistence or lack of persistence of an alteration in the activation during terminal swing and early stance, could answer this question.

Similarly, the pathological character of triceps surae overactivity during the terminal swing phase can be questioned, since it could correspond to an eccentric contraction cushioning the body weight during initial digitigrade contact.

### Limitations of the Study

The first limitation of this study is its retrospective nature, which does not allow prior standardization of the characteristics of subjects, and leads to a lower level of evidence. However, despite the retrospective nature of the study, standardization of clinical and instrumental data collection and triple-blind evaluation of video analysis criteria optimized the reproducibility of measurements. The small cohort together with a low statistical power can make it difficult to demonstrate a statistically significant difference in terms of the *p*-value or confidence interval of effect size. Interpretation of the odds ratio as an indicator of size effect enabled us to move beyond these limitations. Nevertheless, caution should be exercised when extrapolating results (19).

Furthermore, 12 and 35% of the EMG data were uninterpretable for the soleus and medial gastrocnemius, respectively. This could be improved by preprocessing and normalization of the EMG data, as it is difficult to distinguish background noise from permanent overactivity.

The lack of 3D-IGA to assess ADF at the post-MNB stage is also a limitation since video assessment is not the gold standard in terms of kinematic data collection. Only 3 studies discussed the use of an optoelectronic system to compare ankle kinematics after isolated tibial nerve neurotomy in a total of 28 patients (1, 29, 30). They all demonstrated increased ADF during the stance phase and on initial foot contact (29, 30) with one of the studies highlighting increased ADF during the swing phase (1). Despite the limitations, video assessment is the most common method for assessing tibial MNB impact on ADF (11) and has been used after lower limb surgery (5, 6, 31) involving neurotomy, tendon lengthening and/or transfer, with sufficient sensitivity to change to demonstrate improved ADF during stance and swing phases.

## Conclusion

Given that 3D-IGA is used as a preoperative tool to assess gait, it is important to gain a better understanding of the clinical significance of the data provided. This study assessed the predictive value of dynamic EMG patterns obtained during 3D-IGA on tibial MNB outcomes. It confirms the involvement of spastic co-contraction of the triceps surae during early and mid-swing phases of ankle dorsiflexion defects and that the presence of these co-contractions cannot be predicted by clinical data alone. It shows the relevance of tibial MNB to remove spastic calf dystonia and facilitate the assessment of calf contracture. It also underlines the need for complementary work to specifically analyze the TA activation pattern after MNB to clarify the pathological nature of the loss of activation during a terminal swing. Finally, this study allowed us to summarize the advantages and limitations of the different clinical and instrumental evaluation methods for spastic equinus foot and highlight their complementarity.

## Data Availability Statement

The original contributions presented in the study are included in the article/Supplementary Material, further inquiries can be directed to the corresponding author.

## Ethics Statement

Ethical review and approval was not required for the study on human participants in accordance with the local legislation and institutional requirements. Written informed consent for participation was not required for this study in accordance with the national legislation and the institutional requirements. Written informed consent was obtained from the individual(s) for the publication of any potentially identifiable images or data included in this article.

## Author Contributions

CC and DG ensured the administration of the motor nerve blocks. CC and CS contributed to the collection and interpretation of the data and writing and revising the article critically for important intellectual content. EM and DG contributed substantially to the conception and design of the manuscript and the analysis and interpretation of the data. MS ensured acquisition of quantified gait analysis data. EC-L, XD, and PM participated in the analysis and interpretation of data. All authors contributed to drafting and revising the article and approved the final version of the manuscript.

## Conflict of Interest

The authors declare that the research was conducted in the absence of any commercial or financial relationships that could be construed as a potential conflict of interest.

## Publisher's Note

All claims expressed in this article are solely those of the authors and do not necessarily represent those of their affiliated organizations, or those of the publisher, the editors and the reviewers. Any product that may be evaluated in this article, or claim that may be made by its manufacturer, is not guaranteed or endorsed by the publisher.
